# Phyllosphere Arthropods Facilitate Secondary Dispersal of Putative Mycoparasite *Simplicillium*: A Potential Biocontrol Strategy for Soybean Rust

**DOI:** 10.3390/microorganisms13092035

**Published:** 2025-08-31

**Authors:** Takuma Nada, Yasuhiro Ishiga, Izumi Okane

**Affiliations:** 1 Degree Programs in Life and Earth Sciences, College of Science and Technology, University of Tsukuba, 1-1-1-1 Tennodai, Tsukuba 305-8572, Ibaraki, Japan; nada.takuma.xm@alumni.tsukuba.ac.jp; 2 Institute of Life and Environmental Sciences, University of Tsukuba, 1-1-1 Tennodai, Tsukuba 305-8572, Ibaraki, Japan

**Keywords:** ecology, plant disease, biological control, mycoparasite, IPM

## Abstract

Soybean rust, caused by *Phakopsora pachyrhizi*, is a major foliar disease that often escapes fungicide control, necessitating alternative strategies. We investigated whether phyllosphere arthropods, such as mites and thrips, facilitate the secondary dispersal of the mycoparasitic fungus *Simplicillium* under controlled conditions. Detached soybean leaves inoculated with *P. pachyrhizi* were subjected to either arthropod-exposed or arthropod-excluded treatments. *Simplicillium* isolates were significantly more abundant in the presence of arthropods. Molecular identification revealed identical ITS genotypes of *S. lamellicola* from both infected pustules and thrips, indicating vector-mediated fungal transmission. While some *Simplicillium* strains persisted epiphytically without vectors, their spread was minimal. These results highlight a promising approach to enhance the effectiveness of *Simplicillium*-based biocontrol through natural arthropod-mediated dissemination, warranting field validation of this self-disseminating strategy.

## 1. Introduction

Soybean rust, caused by the obligate biotrophic fungus *Phakopsora pachyrhizi*, is one of the most destructive foliar diseases of soybean worldwide, with yield losses exceeding 80% under favorable conditions [1,2]. Chemical fungicides, although widely used, provide only short-term protection and raise environmental and resistance concerns. The rapid emergence of fungicide-resistant pathogen strains underscores the need for sustainable and durable control options [3].

Biological control agents, such as *Bacillus subtilis* and *Metarhizium* spp., have shown promise in reducing rust severity in greenhouse and field trials. However, consistent field-level efficacy remains elusive due to environmental variability and dispersal limitations [4,5]. Other promising strategies include breeding for host resistance, transgenic approaches, and RNA interference technologies [6,7]. However, the complex nature of *P. pachyrhizi*, notably its ability to overcome single resistance genes, poses challenges for achieving durable control [6]. Consequently, an integrated disease management approach compatibility with the arthropod vectors that combines chemical, biological, genetic, and molecular tactics is likely to provide the most effective and long-lasting suppression of soybean rust [3].

*Simplicillium* W. Gams and Zare (*Cordycipitaceae*: Hypocreales) is a fungal genus exhibiting a wide array of ecological lifestyles, including entomopathogenic, mycoparasitic, and endophytic roles [8,9]. Recent work has described several novel species isolated from arthropods, *S. araneae*, *S. coleopterorum*, and *S. formicae*, underscoring the genus propensity for host switching [9,10]. Morphologically, *Simplicillium* species are distinguished by their solitary phialides and variable conidial forms [11,12]. They also synthesize a diverse suite of secondary metabolites with promising pharmacological activities [13]. Importantly, certain *Simplicillium* species have been identified as hyperparasites of plant pathogens *Puccinia striiformis* f. sp. *tritici* (*Pst*) and *P. pachyrhizi*, showing potential for biological control of wheat stripe rust and soybean rust, respectively [14,15].

Recent studies have demonstrated the biocontrol potential of *Simplicillium lamellicola* against *Fusarium graminearum*, the causal agent of Fusarium head blight (FHB). Under both controlled and field conditions, this species markedly reduced disease severity, achieving up to 88% suppression of FHB symptoms and a 77% reduction in field trials. In addition, *S. lamellicola* has been reported to promote wheat seedling growth by increasing shoot length, root length, and biomass [16]. Collectively, these findings underscore the potential of the genus *Simplicillium* as an effective antagonistic agent in crop protection strategies.

In repeated assays using inoculated soybean leaves in growth chambers, *Simplicillium*-like fungi were frequently recovered from rust pustules, sometimes in the presence of mites or thrips. While fungal spores typically disperse via wind, rain splash, or animal vectors [17,18,19] enclosed settings often favor arthropod-mediated transmission [20,21]. Since *Simplicillium* is closely related to *Leptobacillium symbioticum*, previously found in both *P. pachyrhizi* uredinia and arthropods [22], we hypothesize that phyllosphere arthropods may serve as vectors facilitating secondary fungal dispersal.

This study aims to evaluate the role of phyllosphere arthropods in the dissemination of *Simplicillium* under controlled conditions. To evaluate this, the study will first compare *Simplicillium* isolation frequencies between open-access and arthropod-exclusion treatments; second, perform molecular characterization of isolates from plant tissue and arthropods to establish transmission linkages; and third, delineate the ecological traits and dissemination mechanisms of *Simplicillium* species in a growth-chamber context. Elucidating arthropod roles in *Simplicillium* spread will enhance our understanding of fungal ecology and support the development of self-disseminating biological control strategies. Although this study was conducted under controlled conditions, phyllosphere arthropods such as thrips and mites are commonly found on field-grown soybean plants. Herbivorous insects typically move extensively across leaf surfaces during feeding and oviposition, and in doing so, may transport surface-adhering microbes, including fungi, between tissues of the same plant or among different plants. While direct field-based evidence is currently lacking, these ecological interactions support the plausibility of vector-mediated dissemination of *Simplicillium* under natural conditions.

## 2. Materials and Methods

### 2.1. Fungal Strain and Spore Preparation

Strain T1-2 of *P. pachyrhizi* was originally isolated from the soybean cultivar ‘Tachinagaha’ in Japan [23]. It was maintained on living soybean leaves. Urediniospores were harvested into folded paper, air-dried at room temperature for seven days, and stored at −80 °C until use.

### 2.2. Plant Growth Conditions

Soil was prepared by mixing humus and akadama soil (a granular gray volcanic soil) at a 1:3 (*v*/*v*) ratio. The mixture was filled into plastic pots (11.5 cm diameter), into which three seeds of soybean cultivar ‘Enrei’ (Japan Seed Center Co., Nagano, Japan) were sown. Seedlings were grown in a growth chamber at 28 °C under a 16 h light/8 h dark cycle for two weeks.

### 2.3. Inoculum Suspension and Application

Frozen cryotubes of urediniospores were thawed in running water at 39 °C for 1 min. Spores were suspended in 0.05% (*v*/*v*) Tween-20 (Wako Pure Chemical Industries, Ltd., Saitama, Japan) and adjusted to 2.0 × 10^4^ spores/mL using a hemocytometer. The second or third trifoliate leaves of two-week-old seedlings were sprayed on the abaxial surface with the spore suspension using a hand sprayer (model SN 500, FULUPRA Co., Ltd., Tokyo, Japan). Control leaves received 0.05% Tween-20 only. The suspension was sprayed from a distance of approximately 10 cm, continuing until droplets visibly formed and began to drip from the leaflets to ensure thorough coverage. The number of sprays was not predetermined.

### 2.4. Incubation and Detached-Leaf Assay

After spray inoculation, plants were kept in a dark, high-humidity chamber (≥90% RH) for 24 h. Leaves were then detached and placed abaxial side up between laboratory tissue sheets moistened with 40 ppm gibberellic acid (Wako Pure Chemical Industries, Ltd., Saitama, Japan) solution in square Petri dishes (AW2000, EIKEN CHEMICAL Co., Ltd., Tokyo, Japan); half of these dishes were sealed with plastic wrap to assess arthropod involvement. Gibberellic acid was added to prolong leaflet viability during incubation, as it delays senescence in detached leaf tissue. Nine leaflets (three per dish) comprised one replicate, and all were incubated at 23 °C under a 16 h light/8 h dark cycle for three weeks, with gibberellic acid replenished every 5–7 days. This entire experiment was repeated three times under identical conditions.

### 2.5. Isolation of Simplicillium and Arthropods

White fungal colonies appearing on detached leaflets were transferred to low-nutrient Czapek–Dox agar (LCA) amended with 50 mg/L chloramphenicol and incubated at 25 °C in the dark. LCA medium was prepared as described by Miura and Kudo (1970) [24], containing (*w*/*v*): glucose 0.1%, KH_2_PO_4_ 0.1%, MgSO_4_·7H_2_O 0.02%, KCl 0.02%, NaNO_3_ 0.2%, yeast extract 0.02%, and agar 1.3%. Arthropods (mites and thrips) observed on leaflets were crushed with sterile tweezers and similarly plated on LCA. Emerging hyphal tips were transferred to potato dextrose agar (PDA) and incubated at 25 °C in the dark. No mites or thrips were deliberately introduced during the experiment. They likely entered the unsealed treatment dishes naturally from the surrounding environment within the growth chamber. Tentative morphological observations indicated that the thrips belonged to the genus *Thrips*, although no further taxonomic identification was performed.

### 2.6. Molecular Identification by DNA Sequencing

Genomic DNA from each fungal isolate was extracted using a modified Izumitsu et al. (2012) protocol [25]. The internal transcribed spacer (ITS) region was amplified with primers ITS1f_KYO1/ITS4_KYO1 [26] and the large subunit (LSU) region with NL1/NL4 [27]. PCR reactions (15 µL) contained 1 µL DNA, 1.5 µL of each primer (2 µM), 3.5 µL Milli-Q water, and 7.5 µL EmeraldAmp™ PCR Master Mix (Takara Bio Inc., Shiga, Japan). ITS cycling was 95 °C for 3 min; 35 cycles of 95 °C for 30 s, 55 °C for 1 min, 72 °C for 1 min; final extension 72 °C for 10 min. LSU cycling was 94 °C for 3 min; 30 cycles of 94 °C for 30 s, 50 °C for 30 s, 68 °C for 1 min; final extension 68 °C for 7 min. Amplified products were visualized on 1.5% agarose gels stained with Midori Green Direct (Nippon Gene Co., Ltd., Tokyo, Japan), purified with ExoSAP-IT (Thermo Fisher Scientific, Waltham, MA, USA), and Sanger-sequenced by Eurofins Genomics (Tokyo, Japan). Sequences were assembled in BioEdit v7.0.5.3 [28], trimmed for redundancies, and identified via BLASTN 2.16.0+ (BLAST) searches against GenBank.

### 2.7. Statistical Analysis

The experiment was conducted using a completely randomized design with four treatments: Sealed Control, Unsealed Control, Sealed Inoculated, and Unsealed Inoculated. Each treatment included three replicate Petri dishes (*n* = 3). Each Petri dish contained three soybean leaflets, giving a total of nine leaflets per replicate. Data were aggregated at the replicate level (i.e., one Petri dish) for statistical analyses.

The number of leaflets (nine per replicate) from which 24 *Simplicillium* strains identified by molecular identification was statistically evaluated among treatments using one-way ANOVA. Tukey’s Honestly Significant Difference (Tukey’s HSD) test were subsequently performed to assess pairwise differences between treatments. To validate the assumptions of ANOVA, the Shapiro–Wilk test and Levene’s test were performed on model residuals. Additionally, due to the small number of replicates (*n* = 3 per treatment), we also applied a non-parametric Kruskal–Wallis test and Dunn’s test with Bonferroni correction to ensure robustness. The mean values and standard errors were calculated using Excel, and statistical analyses as well as graph generation were performed using Python 3.11.12.

## 3. Results

### 3.1. Development of P. pachyrhizi Uredinia and Associated White Colonies

**Figure 1:**
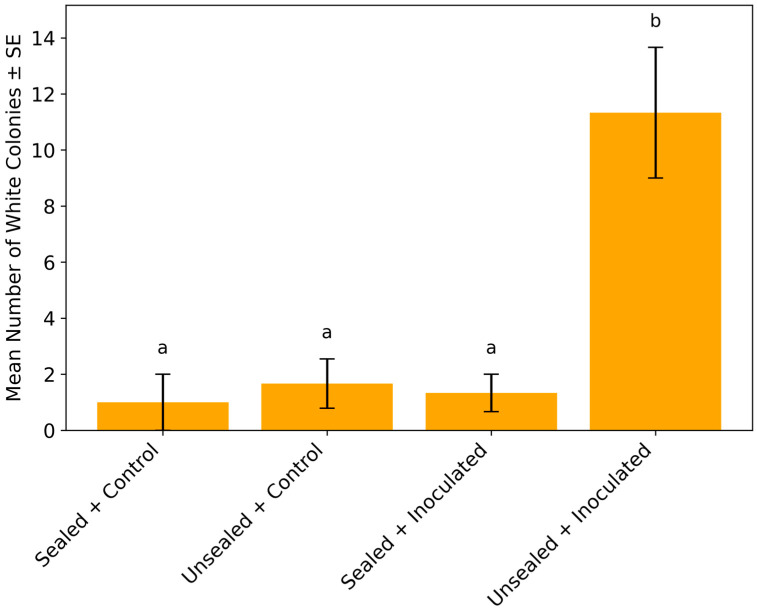
Comparison of *Simplicillium*-like white colony counts across treatments. The unsealed inoculated treatment yielded a mean of 11.3 ± 2.3 white *Simplicillium*-like colonies per replicate, significantly higher than the sealed inoculated (1.3 ± 0.7), unsealed control (1.7 ± 0.9), and sealed control (1.0 ± 1.0) groups (one-way ANOVA, F (3, 8) = 13.08, *p* = 0.002). Tukey’s HSD test (*p* < 0.05) indicated that the unsealed inoculated treatment was significantly different from all other treatments. Different letters (a, b) above the bars denote statistically distinct treatments. Each treatment included three replicates (n = 3).

This analysis clarifies the spatial association between fungal colonies and rust pustules, which is key to understanding potential co-colonization or vector-mediated transfer. Uredinia appeared on detached soybean leaflets 7 days after inoculation in both the unsealed and sealed inoculated treatments. In the unsealed inoculated group, *Simplicillium*-like white colonies co-occurred with mites, thrips and occasional egg-like structures on sori: 7 colonies + arthropods in replicate 1, 12 + arthropods in replicate 2, and 15 + arthropods in replicate 3. A statistical comparison of white colony counts among treatments is presented in Figure 1. The sealed inoculated treatment yielded only two white colonies in replicates 1 and 3, with no arthropods observed.

However, in the third replicate of the sealed control treatment, a dead thrips was found on the square Petri Dish. As the dish contained three leaflets, these were excluded from the dataset, while the six leaflets from the replicate were retained for analysis.

**Figure 2:**
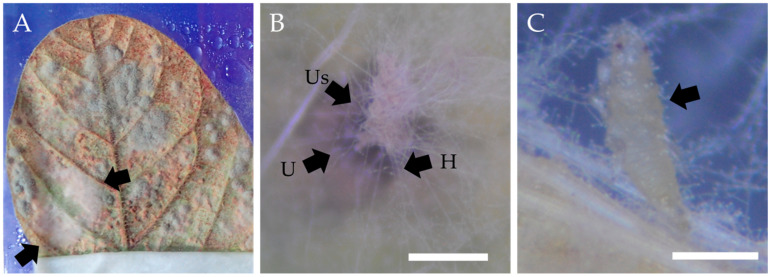
Representative observations of *Simplicillium*-like fungi from the Unsealed Inoculated treatment. (**A**) White, feathery colonies on soybean leaf surfaces (arrows) considered *Simplicillium*-like based on characteristic morphology. (**B**) *Simplicillium*-like hyphae (H) enveloping urediniospores (Us) on a uredinium (U); scale bar = 0.25 mm. (**C**) Thrips larva resembling a member of the genus *Thrips*, partially covered with *Simplicillium*-like hyphae; the larva was still alive at the time of observation; scale bar = 0.25 mm.

In the unsealed control, three white colonies appeared in replicate 1; replicates 2 and 3 harbored mites and thrips (plus two colonies in replicate 3). The sealed control produced three colonies in replicate 1 only, and no arthropods in any replicate. The egg-like structures were observed only briefly during isolation and were neither preserved nor examined in detail; thus, their identity remains unknown. In culture, *Simplicillium* colonies typically produce white, wool-like mycelia, but their appearance on soybean leaf surfaces was sometimes subtle. In this study, fungal isolates were obtained from white, feathery colonies visible to the naked eye, although their visibility varied among samples. A representative example of the colony morphology used for initial recognition and isolation is shown in Figure 2.

### 3.2. Recovery and Selection of Fungal Isolates

**Figure 3:**
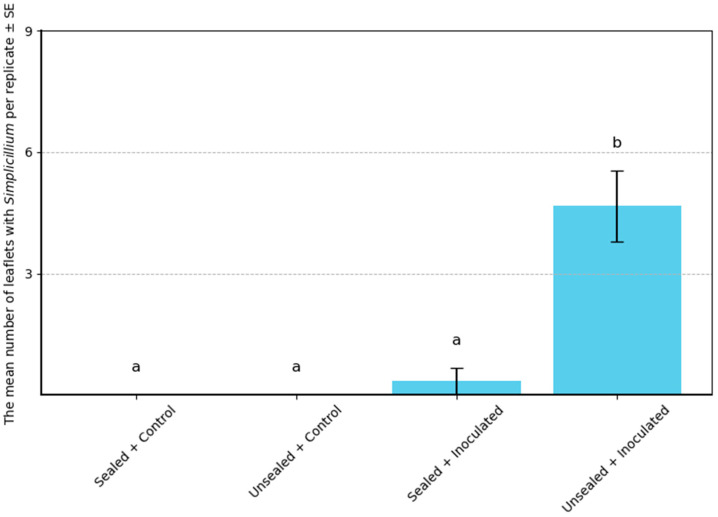
Comparison of the number of *Simplicillium*-covered leaflets across treatments. The unsealed inoculated treatment yielded a mean of 4.7 ± 0.9 leaflets (nine per replicate) from which *Simplicillium* isolates per replicate, significantly higher than the sealed inoculated (0.3 ± 0.6), unsealed control (0.0 ± 0.0), and sealed control (0.0 ± 0.0) groups (one-way ANOVA, F (3, 8) = 23.46, *p* = 0.0003). Tukey’s HSD test (*p* < 0.05) further indicated that the unsealed inoculated group was significantly different from all other treatments. Although Levene’s test supported homogeneity of variance (*p* = 0.951), the residuals deviated from normality (Shapiro–Wilk test, *p* = 0.038). Therefore, we additionally performed a Kruskal–Wallis test, which confirmed significant differences among treatments (*p* = 0.025). A post hoc Dunn’s test with Bonferroni correction showed significant differences between the unsealed inoculated group and both the sealed inoculated and control groups (*p* < 0.05), while the difference between the sealed and unsealed inoculated treatments was marginally non-significant (*p* = 0.13). Different letters (a, b) above the bars indicate statistically distinct groups based on Tukey’s HSD test. Each treatment included three replicates (n = 3).

A total of 46 isolates were recovered from white colonies, arthropods and egg-like structures in the unsealed inoculated treatment; sealed inoculated, unsealed control and sealed control treatments yielded 4, 5 and 3 isolates, respectively. Of the 46 from unsealed inoculated, forty displaying *Simplicillium*-like morphology (white, wooly colonies) were selected for molecular analysis. No *Simplicillium*-like isolates were obtained from either of the control treatments. In addition, some *Simplicillium*-like colonies were observed to entangle urediniospores of *P. pachyrhizi*, suggesting a potential interaction between the fungus and the pathogen (Figure 1). Moreover, several thrips individuals were found partially covered with white fungal material at the time of isolation, indicating that *Simplicillium* may also interact with, or potentially affect, the vectors themselves (Figure 2). Arthropods observed in the unsealed inoculated treatment were photographed at the time of isolation. Based on morphological characteristics visible in the images, the thrips were tentatively identified as belonging to the genus *Thrips*. However, no mites were recovered from which *Simplicillium* was isolated, and thus their role remains unclear in this study. The number of *Simplicillium*-positive leaflets per treatment was quantified to evaluate the frequency of fungal colonization, and is summarized in Figure 3.

### 3.3. Molecular Identification of Simplicillium Species

Molecular identification is essential to confirm species-level identity and to determine whether isolates from different sources (leaflets vs. arthropods) are genetically identical, thus supporting vector-mediated dispersal. BLAST analysis of ITS and LSU rDNA sequences identified 24 isolates as *Simplicillium* (Table 1, Table 2, and Supplementary Table S1). ITS resolved five species: *S. lamellicola*, *S. lanosoniveum*, *S. subtropicum*, and *S. sympodiophorum*; three additional LSU sequences lacked corresponding ITS data and remained undetermined at species level. After discarding duplicates (100% identical ITS), six representative strains (*S. lamellicola* (Sabi11, Th6), *S. lanosoniveum* (3rep-1), *S. subtropicum* (Th5, 3L3-1), *S. sympodiophorum* (Sabi7)) were preserved (Table 3).

In the unsealed inoculated treatment, *S. lamellicola* appeared in all replicates, *S. lanosoniveum* only in replicate 3, *S. subtropicum* in replicates 1 and 3, and *S. sympodiophorum* in replicates 1 and 2 (Table 4). In the sealed inoculated treatment, only *S. lanosoniveum* was recovered (replicate 3). No *Simplicillium* species emerged from either of the control treatments. Sequence comparisons confirmed 100% identity between isolates from leaves versus thrips (for *S. lamellicola* and *S. subtropicum*) and between sealed versus unsealed inoculated *S. lanosoniveum* in replicate 3 (Table 5 and Table 6).

### 3.4. Morphological Characterization of Preserved Isolates

**Figure 4:**
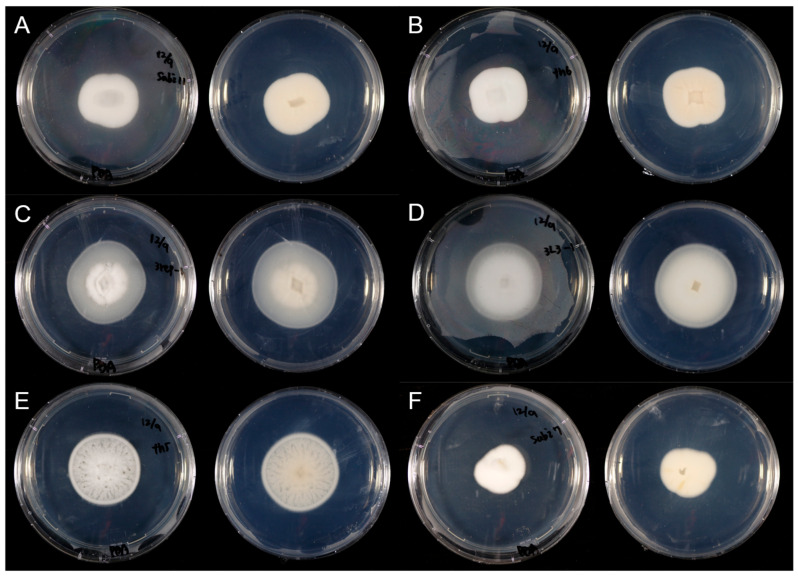
Colony surface (left) and reverse (right) of *Simplicillium* strains cultured on PDA for 10 days at 25 °C. (**A**): *S. lamellicola* (Sabi11), (**B**): *S. lamellicola* (Th6), (**C**): *S. lanosoniveum* (3rep-1), (**D**): *S. subtropicum* (3L3-1), (**E**): *S. subtropicum* (Th5), (**F**): *S. sympodiophorum* (Sabi7).

**Figure 5:**
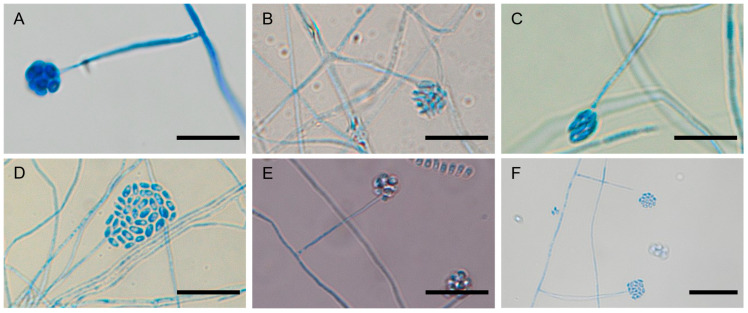
Microscopic images of representative *Simplicillium* isolates recovered from white colonies or thrips. (**A**): *S. lamellicola* (Sabi11), (**B**): *S. lamellicola* (Th6), (**C**): *S. lanosoniveum* (3rep-1), (**D**): *S. subtropicum* (3L3-1), (**E**): *S. subtropicum* (Th5), (**F**): *S. sympodiophorum* (Sabi7). Scale bars: 10 um (**A**–**E**), 20 um (**F**).

All six preserved *Simplicillium* strains formed white, wooly colonies on PDA and produced solitary phialides bearing mucilaginous heads of conidia (Figure 4 and Figure 5). *Simplicillium lamellicola* Sabi11 generated elongated ellipsoidal to fusiform conidia, whereas Th6 yielded exclusively ellipsoidal spores. *S. lanosoniveum* 3rep-1 produced ovoid conidia; *S. subtropicum* 3L3-1 bore ellipsoidal spores; Th5 formed subglobose conidia in mucilage and cylindrical conidia in short chains; and *S. sympodiophorum* Sabi7 produced ellipsoidal to ovoid conidia. Morphological distinctions support molecular findings and help assess phenotypic traits that may influence dispersal efficiency and environmental persistence.

### 3.5. Statistical Analysis

The number of leaflets from which *Simplicillium* was isolated differed among treatments. One-way ANOVA indicated a significant effect of treatment (*p* = 0.012), and Tukey’s HSD test showed that the Unsealed Inoculated treatment had a significantly higher number of *Simplicillium*-positive leaflets than the other treatments (*p* < 0.05). 

Although Levene’s test supported homogeneity of variance (*p* = 9.51), the residuals deviated from normality (Shapiro–Wilk test, *p* = 0.038). Therefore, a Kruskal–Wallis test was also performed, confirming significant differences among treatments (*p* = 0.025). A post hoc Dunn’s test with Bonferroni correction indicated a marginally non-significant difference between Sealed and Unsealed Inoculated treatments (*p* = 0.13).

## 4. Discussion

Our study provides compelling evidence that phyllosphere arthropods, particularly thrips and mites, play a crucial role in facilitating the secondary dispersal of *Simplicillium* fungi under controlled environmental conditions. In treatments where arthropods had access to inoculated soybean leaves, we observed a significantly higher number of white *Simplicillium*-like colonies compared to all other treatments. Statistical analyses (Figure 1 and Figure 3) revealed that both the number of wooly *Simplicillium*-like colonies and the number of leaflets from which *Simplicillium* was successfully isolated were significantly greater in the arthropod-accessible treatment. These findings strongly support the conclusion that arthropods function not merely as passive cohabitants, but as active vectors facilitating fungal dissemination. This finding lays the foundation for understanding how biotic vectors may be harnessed to improve the efficacy of fungal biocontrol agents.

Molecular analysis reinforced the role of arthropods as vectors in *Simplicillium* dispersal. Specifically, both *S. lamellicola* and *S. subtropicum* were isolated from *P. pachyrhizi* uredinia and associated thrips within the arthropod-accessible treatment. Notably, the ITS sequences of *S. lamellicola* isolates from a rust pustule and from a thrip in separate replicates were found to be 100% identical, providing strong molecular support for the transfer of fungal propagules via insect contact. This genetic match provides strong support for a vector-mediated dissemination pathway. In contrast, sealed treatments lacking arthropod access yielded minimal fungal recovery, indicating that passive colonization alone is insufficient for substantial establishment. These results clearly demonstrate that arthropods actively contribute to the spatial dissemination of *Simplicillium* across host tissues.

The differing recovery frequencies among *Simplicillium* species suggest that each possesses distinct ecological strategies for dispersal and establishment. *Simplicillium lamellicola* was consistently isolated across all replicates and from both rust pustules and thrips, implying a more generalist mode of transmission with strong vector association. In contrast, *S. subtropicum* was detected in only two replicates. Despite being recovered from both rust pustules and thrips, this low detection rate suggests a more opportunistic and potentially context-dependent dispersal mechanism. These interspecific differences may reflect variation in spore adhesion properties, vector specificity or compatibility with the cuticular surface of arthropod hosts, or tolerance to phyllosphere conditions. Such ecological divergence aligns with previous reports highlighting the versatility of the genus *Simplicillium*, whose members inhabit diverse niches ranging from insect hosts to plant pathogens [15,29,30,31].

From an applied standpoint, harnessing natural arthropod activity may represent a promising approach to support *Simplicillium*-based biocontrol strategies, although further studies are needed to validate its effectiveness and practical feasibility. In particular, the consistent recovery of *S. lamellicola* from both rust pustules and thrips suggests that this species may be well suited for vector-assisted dissemination in the field. By inoculating fields with *S. lamellicola* or *S. subtropicum*, it may be possible to recruit indigenous phyllosphere arthropods to distribute the biocontrol agent autonomously, thereby reducing the need for repeated applications and enhancing persistence. Meanwhile, species like *S. lanosoniveum*, which were occasionally recovered in the absence of vectors, may serve as background colonizers that provide continuous, albeit lower-level, biocontrol coverage. Integrating these species according to their ecological roles could form the basis of a self-disseminating, multi-strain biocontrol system.

Despite the promising findings, this study was conducted under controlled environmental conditions, which may not fully capture the complexity of field ecosystems. Translating these insights into practical applications requires further investigation into several key aspects. First, the efficiency and specificity of different arthropod taxa as fungal vectors should be quantified under variable environmental conditions. To address this, future experiments should include the defined release of well-identified arthropod species in controlled environments. This would allow for a more precise evaluation of their vector competence and clarify species-specific contributions to *Simplicillium* dispersal. Second, the influence of factors such as temperature, humidity, leaf surface characteristics, and plant phenology on fungal establishment and dispersal must be evaluated. Third, it is critical to determine whether vector-mediated dissemination leads to meaningful suppression of *P. pachyrhizi* infection under agronomic field conditions. Additionally, systematic quantification of the number of arthropods per test unit (e.g., per box or leaflet) is necessary to more precisely evaluate their contribution to fungal dispersal. Although tentative morphological identification of thrips was performed in this study, such ecological interactions must be validated with more controlled and taxonomically resolved data. Addressing these questions will be essential for developing robust, field-adaptable biocontrol strategies that integrate fungal biology, vector ecology, and crop management. Furthermore, future studies should explore how agricultural practices, including pesticide application, and the use of different host plant species or cultivars may influence the dynamics of this plant-fungus-arthropod system.

Finally, while thrips and mites demonstrated clear capacity to disseminate *Simplicillium* under our experimental conditions, we acknowledge that both taxa can also be significant crop pests [32]. This raises important considerations regarding potential trade-offs between vector-mediated dissemination of beneficial fungi and arthropod-induced crop damage. In this context, the relative importance of arthropod herbivory, fungal pathogen damage, and *Simplicillium*-mediated suppression should also be considered. While *P. pachyrhizi* typically imposes greater yield losses in soybean than moderate levels of thrips feeding, exceptions may occur, particularly when arthropods vector additional pathogens such as viruses. Understanding this balance will be essential for designing vector-assisted biocontrol strategies that maximize benefits while minimizing unintended crop damage. We envisage application of these findings within the framework of Integrated Pest Management (IPM), where pest populations are maintained at low to moderate levels and managed rather than eradicated. Under such scenarios, naturally occurring pest arthropods could serve as auxiliary agents of beneficial mycoparasite dispersal without necessitating their intentional augmentation. Nevertheless, assessing the role of beneficial or neutral arthropods present in cropping systems is a valuable next step. Our current aim was to determine whether naturally occurring phyllosphere arthropods, regardless of pest status could facilitate *S. subtropicum* spread under controlled laboratory conditions, as a first step toward evaluating feasibility under more complex field settings. Future research should therefore include side-by-side assessments of pest and beneficial arthropod taxa to optimize vector-assisted biocontrol strategies while minimizing agronomic risks.

Furthermore: the reliability and effectiveness of *Simplicillium* as a biocontrol agent may be influenced by whether its occurrence is spontaneous or the result of a deliberate release. At present, little is known about the consistency of *Simplicillium* establishment and dispersal when it arises naturally in cropping systems, and its interactions with arthropod vectors remain poorly understood. This uncertainty highlights the need for future studies to evaluate these dynamics under both controlled and field conditions, with particular emphasis on whether arthropod-mediated spread can achieve effective and reproducible suppression of *Phakopsora pachyrhizi*.

## Figures and Tables

**Table 1:** Number of *Simplicillium* strains isolated from wooly white colonies.

Treatment	Replicate1	Replicate2	Replicate3
Unsealed + Inoculated	4	5	11
Sealed + Inoculated	0	0	1
Unsealed + Control	0	0	0
Sealed + Control	0	0	0

**Table 2:** Number of *Simplicillium* strains isolated from arthropods.

Treatment	Replicate1	Replicate2	Replicate3
Unsealed + Inoculated	1	0	2
Sealed + Inoculated	0	0	0
Unsealed + Control	0	0	0
Sealed + Control	0	0	0

**Table 3:** *Simplicillium* isolates and its accession numbers.

Isolate ID	Species	Resource	Region	Accession Number
Sabi11	*Simplicillium lamellicola*	White colony	ITS	PX113195
Th6	*S. lamellicola*	Thrips	ITS	PX113194
3rep1	*Simplicillium lanosoniveum*	White colony	ITS	PX113198
Th5	*Simplicillium subtropicum*	Thrips	ITS	PX113197
3L3-1	*S. subtropicum*	White colony	LSU	PX113200
Sabi7	*Simplicillium sympodiophorum*	White colony	ITS	PX113199

**Table 4:** *Simplicillium* isolates derived from wooly white colonies and arthropods in each treatment and replicate.

Species	UI R1	UI R2	UI R3	SI R1	SI R2	SI R3	UC R1	UC R2	UC R3	SC R1	SC R2	SC R3
*S. lamellicola*	✓	✓	✓	-	-	-	-	-	-	-	-	-
*S. lanosoniveum*	-	-	✓	-	-	✓	-	-	-	-	-	-
*S. subtropicum*	✓	-	✓	-	-	-	-	-	-	-	-	-
*S. sympodiophorum*	✓	✓	-	-	-	-	-	-	-	-	-	-

✓: *Simplicillium* isolated; -: not isolated. UI: Unsealed Inoculated, SI: Sealed Inoculated, UC: Unsealed Control, SC: Sealed Control.

**Table 5:** *Simplicillium* isolates derived from white colonies in each treatment and replicate.

Species	UI R1	UI R2	UI R3	SI R1	SI R2	SI R3	UC R1	UC R2	UC R3	SC R1	SC R2	SC R3
*S. lamellicola*	✓	✓	-	-	-	-	-	-	-	-	-	-
*S. lanosoniveum*	-	-	✓	-	-	✓	-	-	-	-	-	-
*S. subtropicum*	✓	-	✓	-	-	-	-	-	-	-	-	-
*S. sympodiophorum*	✓	✓	-	-	-	-	-	-	-	-	-	-

✓: *Simplicillium* isolated; -: not isolated. UI: Unsealed Inoculated, SI: Sealed Inoculated, UC: Unsealed Control, SC: Sealed Control.

**Table 6:** *Simplicillium* isolates derive from arthropods in each treatment and replicate.

Species	UI R1	UI R2	UI R3	SI R1	SI R2	SI R3	UC R1	UC R2	UC R3	SC R1	SC R2	SC R3
*S. lamellicola*	✓	-	✓	-	-	-	-	-	-	-	-	-
*S. lanosoniveum*	-	-	-	-	-	-	-	-	-	-	-	-
*S. subtropicum*	-	-	✓	-	-	-	-	-	-	-	-	-
*S. sympodiophorum*	-	-	-	-	-	-	-	-	-	-	-	-

✓: *Simplicillium* isolated; -: not isolated. UI: Unsealed Inoculated, SI: Sealed Inoculated, UC: Unsealed Control, SC: Sealed Control.

## Data Availability

The original contributions presented in this study are included in the article/supplementary material. Further inquiries can be directed to the corresponding authors.

## Supplementary Materials

The following supporting information can be downloaded at: https://www.mdpi.com/article/10.3390/microorganisms13092035/s1. Supplementary Table S1. BLAST results of ITS and LSU rDNA sequences of all 24 *Simplicillium* isolates. The table includes isolate ID, region, species, accession number, resource.
